# Mountain goat survival and mortality during a period of increased puma abundance in the Black Hills, South Dakota

**DOI:** 10.7717/peerj.9143

**Published:** 2020-05-29

**Authors:** Chadwick P. Lehman, Eric M. Rominger, Brady Y. Neiles

**Affiliations:** 1 South Dakota Department of Game, Fish, and Parks, Custer, SD, United States of America; 2 New Mexico Department of Game and Fish, Santa Fe, NM, United States of America

**Keywords:** Mountain goat, Oreamnos americanus, Puma, Puma concolor, Survival, Mortality, Black hills, Realized niche, Alternate prey

## Abstract

We investigated survival and cause-specific mortality for a mountain goat (*Oreamnos americanus*) population during a period when the puma (*Puma concolor*) population was growing in the Black Hills, South Dakota, 2006–2018. We obtained survival data from 47 adult goats (*n* = 33 females, *n* = 14 males). Annual survival varied from 0.538 (95% CI [0.285–0.773]) to 1.00 (95% CI [1.00–1.00]) and puma predation was the primary cause-specific mortality factor over a 12-year period. Cumulative hectares of mountain pine beetle (*Dendroctonus ponderosae*) disturbance was a covariate of importance (*w*_*i*_ = 0.972; *β* = 0.580, 95% CI [0.302–0.859]) influencing survival. To our knowledge, this is the first account of puma being the primary mortality factor of mountain goats over a long-term study. The Black Hills system is unique because we could examine the expanded realized niche of puma in the absence of other large carnivores and their influence on mountain goats. We hypothesize that puma were being sustained at higher densities due to alternate prey sources (e.g., white-tailed deer; *Odocoileous virginianus*) and this small population of mountain goats was susceptible to predation by one or several specialized puma in the Black Hills. However, we also hypothesize a changing landscape with increased tree mortality due to insect infestation provided conditions for better predator detection by goats and increased survival. Alternatively, open canopy conditions may have increased understory forage production potentially increasing mountain goat survival but we did not evaluate this relationship. Survival and mortality rates of mountain goats should continue to be monitored as this small population may be highly susceptible to population declines due to slow growth rates.

## Introduction

Dynamics of ungulate-predator systems can be difficult to disentangle because of the confounding effects of weather, alternate prey sources, and the interactions of multiple predator species (Bergerud & Ballard, 1988; Boutin, 1992; Rominger et al., 2004; Festa-Bianchet & Côté, 2008). Predator populations can facilitate ecosystem change by altering abundance or behavior of prey (Schmitz et al., 2008). In some cases, the reduction or extinction of one prey species may be controlled by a predator that is augmented by an abundant, alternate prey species (DeCesare et al., 2010). In ecological theory ‘apparent competition’ is the indirect interaction between (at least) two prey species and a shared predator (Holt, 1977). A decline in one species may coincide with an increase in the other and the conservation of large mammals depends on our understanding this interaction in small populations (Morris & Doak, 2002). Unfortunately, there is little information on the unpredictable nature of predation by individual specialist predators on small ungulate populations (Vazquez-Dominguez, Ceballos & Cruzado, 2004; Festa-Bianchet et al., 2006). Whether it be by a specialist predator, or by apparent competition with increased alternate prey, some isolated or endangered ungulate populations have been unable to recover (Festa-Bianchet, 1991; Kinley & Apps, 2001; Festa-Bianchet et al., 2006).

The dynamics of predator–prey ecology as it pertains to mountain goats (*Oreamnos americanus*) is very different from most mountain ungulate populations because their generally small population size does not provide a sufficient prey base to sustain most predators (Festa-Bianchet & Côté, 2008). Mountain goats rely on precipitous escape terrain to minimize predation risk and in the Black Hills rugged habitats are typically granite outcroppings (South Dakota Department of Game, Fish and Parks, 2018). Unlike cervids, their affinity for using precipitous escape terrain makes it much more difficult for coursing predators to prey upon them, so a specialized hunting technique would be required (Côté & Festa-Bianchet, 2003). A stalking predator such as puma (*Puma concolor*) could become an effective predator of mountain goats, particularly if mountain goats are predictable in using habitats where they are more vulnerable over time (Festa-Bianchet & Côté, 2008). A few specialized predators could have a substantial impact on small mountain goat populations because they are vulnerable to decline because of their low rates of population recruitment (Bailey, 1991; Festa-Bianchet, Urquhart & Smith, 1994). Most mountain goat populations are too small to provide a prey base for a population of predators, and a single puma that specialized on preying on mountain goats could have a strong influence on a local herd (Côté & Festa-Bianchet, 2003). Therefore, it has been hypothesized that such effects of predation on mountain goat population dynamics may be density independent (Côté & Festa-Bianchet, 2003).

Predation on mountain ungulates, hypothetically, has been influenced by a change in the apex predator guild following the extirpation of wolves (*Canis lupus*) and grizzly bears (*Ursus arctos*) in many areas of their former native range (Rominger, 2018). Following the extirpation of wolves in the western United States in the early 1900’s, the formerly subordinate puma became the dominant predator of mountain ungulates in many ecosystems (Boyd & Neale, 1992; Kortello, Hurd & Murray, 2007; Ruth et al., 2011; Elbroch et al., 2015).  Puma have been documented as the primary predator of ungulates including mule deer (*Odocoileus hemionus*) (Logan & Sweanor, 2001), elk (*Cervus elaphus*) (Rearden, Anthony & Johnson, 2011; Lehman, Rota & Millspaugh, 2017a), woodland caribou (*Rangifer tarandus*) (Kinley & Apps, 2001), and bighorn sheep (*Ovis canadensis*) (Rominger & Weisenberger, 2000; Rominger et al., 2004). The ecological shift of the apex predator guild from a coursing Canid predator to a stalking Felid predator has contributed to the decline of some ungulate populations such as woodland caribou (Compton, Zager & Servheen, 1995), Sierra Nevada bighorn sheep (Wehausen, 1996), and New Mexico desert bighorn sheep (Goldstein & Rominger, 2012).  The ability of puma to prey switch from more numerous sympatric ungulates, primarily deer (*Odocoileus spp*.) or domestic cattle (*Bos Taurus*), contributes to declining populations of the more rare ungulate (Lehman, Rota & Millspaugh, 2017a; Rominger, 2018).  It is hypothesized that many mountain ungulates have fallen into an ecological trap following the expansion of the realized niche of pumas (Hutchinson, 1957; Dwernychuk & Boag, 1972; Rominger, 2018).  The current facilitation of wolf and grizzly bear recovery may have profound effects on the persistence of puma and its role as a dominant predator in some mountain ungulate communities.

Puma need to stalk within a short distance from prey in order to make a successful kill; dense vegetation is often used to cover their approach before attacking (Logan & Irwin, 1985; Koehler & Hornocker, 1991; Laundre & Hernandez, 2003; Lehman et al., 2017b). Transition zones from cover or escape terrain areas to foraging areas have been hypothesized to provide areas where puma may have greater success of killing prey (Laundre & Hernandez, 2003). When mountain goats cross transition zones that are heavily forested they may be highly susceptible to puma predation (Côté & Festa-Bianchet, 2003; Laundre & Hernandez, 2003). It has been hypothesized that risk of predation by puma on mountain goats is higher in areas with trees which provide cover for ambush predators (Côté & Beaudoin, 1997; Côté & Festa-Bianchet, 2003).

Mountain goats were introduced into the Black Hills in 1924 and this heavily forested system is unlike the open alpine habitats typical of their native range (South Dakota Department of Game, Fish and Parks, 2018). Surveys indicated our study population to be small and slow growing from 2000–2018. Mountain goat counts have varied from a high of ∼130 in 2016–18 to < 60 in 2009–11 with an average annual growth rate of 1.02 (95% CI [0.88–1.16]) (South Dakota Department of Game, Fish and Parks, 2018). Managers have struggled to understand the potential causes for this population’s limited population growth, but a hypothesis is that mountain goats may have fallen into an ecological trap by puma predation in the Black Hills. Both wolves and grizzly bears were extirpated from the Black Hills allowing the once subdominant puma to become dominant (Mattson & Merrill, 2002; Ripple, Wirsing & Letnic, 2013). We also hypothesize that the unique vegetation conditions of the Black Hills may impede the ability of mountain goats to detect predators because dense ponderosa pine (*Pinus ponderosa*) stands reduce visibility while also providing stalking cover for puma. The Black Hills offered a unique opportunity to examine mountain goat survival and mortality in a system dominated by a single large carnivore. Our primary objectives were to: (1) determine annual survival rates of mountain goats; and (2) quantify cause-specific mortality.

## Materials & Methods

### Study Area

**Figure 1:**
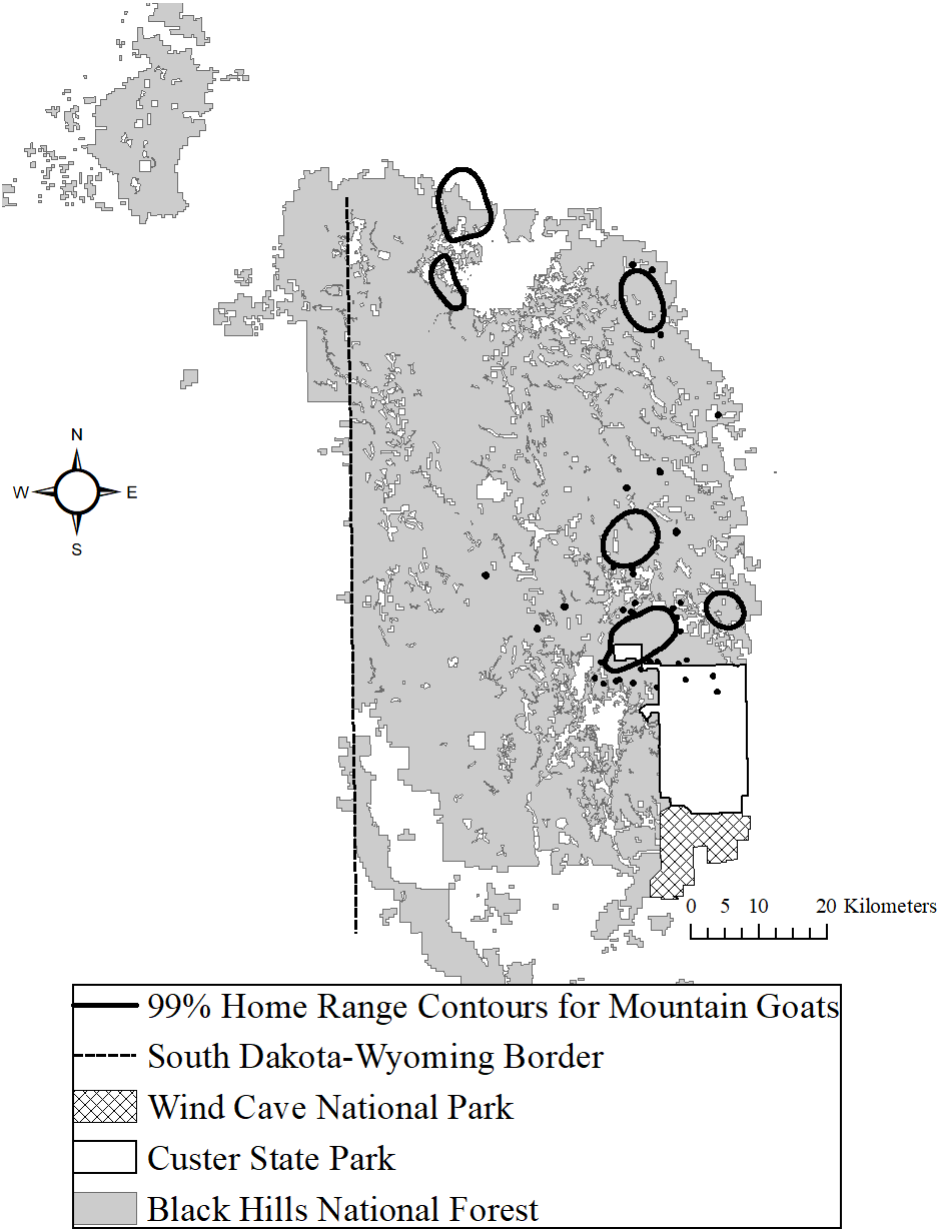
The Black Hills, South Dakota study area where we studied mountain goats, 2006–2018. We provide the spatial distribution of goats using locations with 99% Brownian bridge movement model contours.

The study area was located in Custer, Pennington, and Lawrence counties spanning the Black Hills, South Dakota, USA (Flint, 1955) and was primarily public land (Fig. 1). Elevation ranges from 1,186 m to 2,208 m above mean sea level. Mean annual precipitation ranged from 52–54 cm and mean annual temperature ranged from 6−9 °C across the study area (National Climatic Data Center, 2015). Forests were dominated by ponderosa pine and some high elevation areas also had intermixed spruce (*Picea glauca*) and aspen (*Populus tremuloides*; (Larson & Johnson, 1999). Precipitous terrain included granite outcroppings and these geologic features occurred primarily near the highest elevation of Black Elk Peak (Redden, Norton & McLaughlin, 1982). Potential nonhuman predators of mountain goats included puma, bobcats (*Lynx rufus*), and coyotes (*Canis latrans*).

### Capture and handling

We radiomarked study animals from a small population of mountain goats which ranged in size from approximately 50–130 animals (Table 1). We captured adult mountain goats (≥3 years of age) using net-guns fired from helicopters and from the ground (Cadsand, Jex & Gillingham, 2010); we also captured mountain goats using clover traps baited with salt during October through March 2000–2016 (Clover, 1956; Cadsand, Jex & Gillingham, 2010). We fitted captured goats with very high frequency (VHF) telemetry collars (Telonics, Mesa, AZ, USA; Advanced Telemetry Systems, Isanti, MN, USA). Captured goats were aged by counting horn annuli (Brandborg, 1955; Stevens & Houston, 1989). We subsequently classified individuals into 2 *a priori* age classifications chosen to correspond with stages of senescence and producing young (Côté & Festa-Bianchet, 2003; White et al., 2011). We classified goats as adult (3–9 yrs) and old adult (10+ yrs). We did not mark any goats that were yearling or 2 years old (i.e., subadult) so we only evaluated 2 age classes. We monitored most individuals over multiple years. All handling, marking, and monitoring procedures were approved by the South Dakota Department of Game, Fish, and Parks (Permit Numbers 1–3).

### Survival and mortality

We used daily survival analysis (Nest Survival Module in Program MARK, version 9.0; White & Burnham, 1999; Cooch & White, 2009) via RMark (Laake, 2013) in Program R (Team, 2019) to examine specific hypotheses regarding how age, gender, and mountain pine beetle disturbance influenced mountain goat survival. This approach uses generalized linear modeling with the use of the logit link function as it is the natural link for the binomial distribution (McCullagh & Nelder, 1989; Rotella, Dinsmore & Shaffer, 2004)*.* We hypothesized that old age can negatively influence survival, particularly when goats are ≥10 years of age (Festa-Bianchet & Côté, 2008). We hypothesized that males will have poorer survival than females (Festa-Bianchet & Côté, 2008). It has been hypothesized that risk of predation by puma on mountain goats is higher in areas with trees which provide cover for ambush predators (Côté & Beaudoin, 1997; Côté & Festa-Bianchet, 2003). We hypothesize that cumulative hectares of mountain pine beetle (*Dendroctonus ponderosae*) disturbance will create more open conditions for mountain goats to visually detect predators (see description of how we developed this covariate below). We ranked our competing models using Akaike’s Information Criterion (Burnham & Anderson, 2002). We considered models differing by ≤2 AIC_c_ as alternatives to the selected model but preferred the simplest model (Burnham & Anderson, 2002). We based our conclusions on parameter estimates from the best model.

Telemetry on individuals was conducted 2–3 times weekly to determine survival status (Broecher, 2013). We evaluated annual periods of survival based on the biological period of when most mountain goats are born, which occurs from 1 June–31 May of each year (White et al., 2011). Once a mortality signal was detected we determined cause-specific mortality of goats immediately using several diagnostics. We necropsied the carcass immediately and scrutinized mortality sites for predator sign. We classified cause of mortality as: (1) predation when evidence at the mortality site indicated that the goat had been alive when attacked (e.g., hemorrhaging); (2) fall; (3) drowning; and (4) unknown if the cause of mortality could not be determined. We further assigned predation-caused mortalities to species of predator based on characteristics of predator kills (O’Gara, 1978). We investigated the area for cache sign, drag marks, scat, and hair (Lehman, Rota & Millspaugh, 2017a). We necropsied goat remains, looking for signs of hemorrhaging and bite marks, and measured bite marks to the nearest mm.

We estimated cause-specific mortality rates using cumulative incidence functions (CIF; (Heisey & Patterson, 2006). We used the “mort” package of Program R (Sargeant, 2020) to estimate CIFs, which allowed the estimation of cause-specific mortality in the presence of competing mortality factors (Heisey & Patterson, 2006). Competing factors occur when an animal is exposed to ≥1 potential cause of mortality, and the incidence of one event prevents others from occurring.

### Evaluation of habitat change

We first estimated 99% contour home ranges with Brownian bridge movement models (BBMMs), using a 50 m grid size (Horne et al., 2007), implemented with the ‘BBMM’ package (Nielson, Sawyer & McDonald, 2014) in program R (ver. 3.3.2, 2019, <http://www.r-project.org >) with VHF transmitters that were visually located 2–3 times weekly. We estimated home ranges annually from 1 June–31 May (Fig. 1). We then overlaid the 99% contours with the Black Hills National Forest (BHNF) Forest Service Vegetation (FSVEG) GIS coverage (BHNF, unpublished data) of mountain pine beetle infested trees. This coverage was developed using a combination of aerial surveys, NAIP data, and aerial photographs that were digitized into ArcGIS from 2006–2018 (Backsen & Howell, 2013). Cumulative hectares of mountain pine beetle disturbance was used as a covariate in mountain goat survival.

### Abundance estimates

We obtained abundance estimates of mountain goats using helicopter surveys. A sightability model was used to estimate population size using radio-collars and the mean detection rate from several flights conducted from 2006–2013; from 2014–2018, a Poisson log-normal mark-resight estimate was used to estimate population size from radio-collared mountain goats in 2-year intervals (Broecher 2013, South Dakota Department of Game, Fish and Parks, 2018). Abundance estimates occurred in the core area of the mountain goat range (South Dakota Department of Game, Fish and Parks, 2018). We obtained abundance estimates of puma (which includes kittens) using the Lincoln-Peterson method with radiomarked or DNA marked individuals and hunter harvest; abundance estimates occurred within the Black Hills Fire Protection District (South Dakota Department of Game, Fish and Parks, 2019).

## Results

We obtained survival data from 47 adult (≥3 years of age) individuals (*n* = 33 females, *n* = 14 males). Pooled annual survival probability varied from 0.538 (95% CI [0.285–0.773]) to 1.000 (95% CI [1.000–1.000]) from 2006–2018 (Table 2). Mean annual survival was 0.902 (95% CI [0.825–0.979]) from 2006–18. Puma predation was the largest percentage of cause specific mortality (*n* = 8 of 17; Table 3). In 2007, during a year of low mountain goat abundance (n = ∼50–70) and poor survival (0.538), puma predation (0.289) was the leading cause of mortality for radio-marked mountain goats. The puma population increased substantially from 2006 to 2012 in the Black Hills and has remained relatively stable through 2018 (Table 4).

**Table 1:** Abundance^a^ estimates of mountain goats in the Black Hills, South Dakota 2006–2018.

Year	Abundance estimate	95% CI
2006	70	[61, 79]
2007	62	[53, 71]
2008	71	[60, 81]
2009	56	[48, 65]
2010	76	[64, 88]
2011	55	[46, 63]
2012	104	[89, 120]
2013	111	[95, 127]
2014	121	[99, 207]
2016	133	[106, 236]
2018	135	[95, 373]

**Notes.**

a Using helicopters, a sightability model was used to estimate population size using radio-collars and the mean detection rate from several flights conducted from 2006–2013. Using helicopters from 2014–2018, a Poisson log-normal mark-resight estimate was used to estimate population size from radio-marked mountain goats in 2-year intervals. Abundance estimates occurred in the core area of the mountain goat range.

**Table 2:** Apparent annual survival probability of mountain goats in the Black Hills, South Dakota 2006–2018.

Year	*n*	Survival	95% CI
2006	18	0.652	[0.360, 0.862]
2007	15	0.538	[0.285, 0.773]
2008	13	0.925	[0.618, 0.990]
2009	17	1.000	[1.000, 1.000]
2010	17	0.821	[0.569, 0.941]
2011	14	0.925	[0.615, 0.990]
2012	12	1.000	[1.000, 1.000]
2013	15	1.000	[1.000, 1.000]
2014	15	1.000	[1.000, 1.000]
2015	15	0.934	[0.652, 0.991]
2016	14	1.000	[1.000, 1.000]
2017	14	0.927	[0.622, 0.990]
2018	13	1.000	[1.000, 1.000]

**Table 3:** Estimates of annual mortality rates with 95% confidence intervals and number of deaths observed (n) for mountain goats monitored in the Black Hills, South Dakota 2006–2018. Years not listed below had annual survival of 1.0 with no mortalities.

Year		
Mortality factor	*n*	Mortality rate^a^	95% CI
2006			
Puma	1	0.071	[0.000, 0.197]
Old age	1	0.077	[0.000, 0.211]
Fall	1	0.125	[0.000, 0.327]
Drowning	1	0.077	[0.000, 0.211]
2007			
Puma	4	0.289	[0.052, 0.495]
Unknown	2	0.175	[0.000, 0.369]
2008			
Unknown	1	0.077	[0.000, 0.211]
2010			
Puma	2	0.118	[0.000, 0.258]
Fence	1	0.059	[0.000, 0.164]
2011			
Fence	1	0.071	[0.000, 0.197]
2015			
Puma	1	0.067	[0.000, 0.185]
2017			
Unknown	1	0.071	[0.000, 0.197]

**Notes.**

a We estimated cause-specific mortality using cumulative incidence functions (CIF; Heisey & Patterson, 2006).

**Figure 2:**
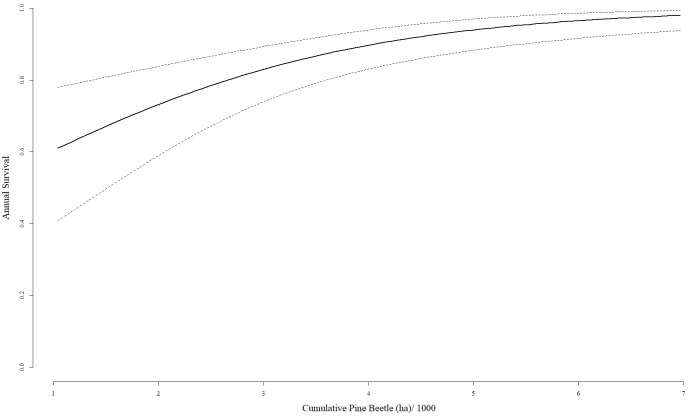
Survival predictions and 95% confidence intervals based on the cumulative hectares of mountain pine beetle mortality model for mountain goats. Estimates are based on annual survival data collected in the Black Hills, South Dakota, USA, June 2006–May 2018.

The top-ranked model explaining annual mountain goat survival included cumulative hectares of mountain pine beetle disturbance (β = 0.580, 95% CI [0.302–0.859]; Table 5). Cumulative hectares of mountain pine beetle tree mortality had a positive relationship on mountain goat survival (Fig. 2). Other model coefficients from competing models evaluated included age class (β = 0.333, 95% CI [−0.772–1.439]), gender (β = 0.471, 95% CI [−0.585–1.526]), and year (12 different β coefficients and all of the CIs overlapped 0).

**Table 4:** Abundance estimates^a^ and harvest of puma in the Black Hills, South Dakota 2006–2018.

Year	Abundance estimate	Abundance method	95% CI	Harvest (*n*)
2006	135	Radiomarked	[90, 180]	13
2007	155	Radiomarked	[113, 197]	15
2008	386	Radiomarked	[223, 549]	17
2009	200	Radiomarked	[149, 251]	26
2010	259	Radiomarked	[161, 359]	40
2011	395	Radiomarked	[154, 639]	47
2012	314	Radiomarked	[179, 450]	73
2013	229	Radiomarked	[154, 305]	61
2014	276	DNA	[146, 411]	53
2015	245	DNA	[131, 365]	43
2016	260	DNA	[138, 390]	41
2017	300	DNA	[114, 495]	30
2018	532	DNA	[111, 970]	31

**Notes.**

a Puma abundance, which includes kittens, was estimated using the Lincoln-Peterson method with radiomarked or DNA marked individuals and hunter harvest. Abundance estimates occurred within the Black Hills Fire Protection District.

**Table 5:** Results of model selection for survival of mountain goats in the Black Hills, South Dakota 2006–2018. Competing models included the covariates age class, gender, year, and cumulative hectares of mountain pine beetle disturbance (beetle).

Survival Models	AIC_c_	ΔAIC	K	*w*_*i*_
Beetle^a^	296.370	0.000	2	0.951
Year	303.515	7.145	13	0.027
Age class^b^ + beetle^a^ + year	304.949	8.579	15	0.013
Gender + age class + beetle^a^ + year	306.820	10.450	16	0.005
Gender + beetle^a^ + year	307.276	10.906	15	0.004
Intercept model	314.726	18.356	1	<0.001
Gender	315.993	19.624	2	<0.001
Age class^b^	316.390	20.020	2	<0.001

**Notes.**

a Cumulative hectares of mountain pine beetle disturbance found within 99% Brownian bridge movement model home ranges of mountain goats.

b Age classes were adult (3–9 years old) and old adult (≥10 years old).

## Discussion

Collecting demographic data and using marked individuals of known age is important for assessing population dynamics of long-lived species (Oli, 2003); however, such information is rarely available (Hamel, Côté & Festa-Bianchet, 2006). Population dynamics of mountain goats are poorly understood and vary greatly across their range (Festa-Bianchet, Urquhart & Smith, 1994; Côté & Festa-Bianchet, 2003). Sensitivity analysis indicated survival of adult females is very important for population growth of mountain goats, particularly for older aged females (5+ years and older; (Hamel, Côté & Festa-Bianchet, 2006). In our study, survival estimates were lower from 2006–2010 (0.79) and improved during 2011–2018 (0.97) with overall average survival (0.90) being similar to that reported for annual survival of adult females (≥2 years of age) across their range (Smith, 1986a; Festa-Bianchet and Côté, 2008).

In the absence of other large carnivores, puma predation was the dominant source of mortality on mountain goats in the Black Hills. To our knowledge this is the first account of puma being the primary mortality factor of mountain goats over a long-term study. From 2006–2010 the Black Hills mountain goat population may have been in an ecological trap where adaptations derived over time were no longer adaptive. Our case study is similar to other systems where a mountain goat population is too low to provide a consistent prey base for a population of predators, and a single puma that specialized on preying on mountain goats could have a strong impact on a local herd (Côté & Festa-Bianchet, 2003). It has been hypothesized that such predation on mountain goat population dynamics may be density independent (Côté & Festa-Bianchet, 2003). In Alberta, with a more complex apex predator guild, wolves and grizzly bears were 79% of the cause-specific predation mortality of mountain goats, whereas puma were subdominant at 21% of the predation mortality Festa-Bianchet & Côté (2008).

Following the introduction of mountain goats in the early 1920s predation was limited to bobcats and coyotes which are considered inconsequential predators of mountain goats. However, after decades of protection and apex predator management programs in North America, puma have successfully recovered from near extirpation in the early 1900s, leading to reestablishment in many areas including the Black Hills (Berger & Wehausen, 1991; Boyce & Byrne, 2009). Since being listed as a South Dakota state-threatened species in 1978, pumas have increased substantially and the first hunting season of puma occurred in 2005 (South Dakota Department of Game, Fish and Parks, 2019). During 2000-01 mountain goats were certainly exposed to a large stalking carnivore capable of specialized predation in precipitous terrain as visual observations of puma were being reported in the Black Elk Peak area.

Simultaneous with the puma increase, the Black Hills was also undergoing a habitat change of increased ponderosa pine tree density leading to less visibility near escape terrain up until 2006 (Shepperd & Battaglia, 2002; Battaglia, Smith & Shepperd, 2008). Risk of predation by puma is hypothesized to be higher in areas with trees which provide cover for ambush predators (Festa-Bianchet, Urquhart & Smith, 1994; Côté & Beaudoin, 1997). When moving between areas of escape terrain through transition zones that are heavily forested, mountain goats normally use traditional and well-marked trails and often run through areas that are heavily forested (Côté & Festa-Bianchet, 2003). A study of kill sites indicated greater density of trees led to increased susceptibility of elk to puma predation in the Black Hills (Lehman et al., 2017b).

Vegetation conditions changed following a mountain pine beetle epidemic that peaked in the Black Hills in 2012 (Graham et al., 2016). From 2006 to 2012 86% of the mountain goat range had been impacted by mountain pine beetle disturbance causing substantial tree mortality (K Allen, 2016, pers. comm.). Annual survival of mountain goats increased to ≥0.97 following the beetle epidemic from 2011–2018. We hypothesize that the beetle epidemic increased the ability of mountain goats to detect predators due to the increased visibility provided by a reduction in tree cover, particularly in the travel corridors between granite outcroppings where previously dense stands of ponderosa pine were now dead.

It has been hypothesized that an increasing, inversely density dependent predation rate could occur, given a declining prey population and a stable puma population (Rominger et al., 2004; DeCesare et al., 2010; Lehman, Rota & Millspaugh, 2017a). These effects may manifest given the ability of puma to readily switch to alternate prey, ultimately subsidizing the puma population (Rominger et al., 2004; DeCesare et al., 2010). We hypothesize that in the Black Hills, mountain goat abundance did not increase in part because the puma population has been sustained by white-tailed deer (*Odocoileous virginianus*) as primary prey (Lehman, Rota & Millspaugh, 2017a). White-tailed deer are abundant in our study area at a population estimate of ∼51,000 individuals in 2016 (South Dakota Department of Game, Fish and Parks, 2017). A study of puma diets in the Black Hills indicated deer (*Odocoileus spp*.) comprised the majority of puma diets (83%), and white-tailed deer were the dominant species (63% of total diet; Smith, 2014). Similar patterns of elevated puma predation on less abundant woodland caribou (*Rangifer tarandus caribou*) and mule deer (*O. heminous*) were observed in areas with abundant sympatric white-tailed deer (Kinley & Apps, 2001; Robinson, Wielgus & Gwilliam, 2002).

Additional factors may also explain low abundance and slow population growth for this population. Late primiparity by females (i.e., mean age for producing kids starts at 4.9 years) and low recruitment (Adams & Bailey, 1982; Swenson, 1985; Smith, 1986b; Festa-Bianchet, Urquhart & Smith, 1994; Côté & Festa-Bianchet, 2001) characterize mountain goat reproduction. Survival of kids can provide some recruitment to one year of age (>55%; Smith, 1976; Festa-Bianchet & Côté, 2008) but there are very few 4+ year old individual females having kids from year to year due to small population size.

Our results of older aged goats (≥10 years of age) and males not having decreased survival contradicts previous research (Festa-Bianchet & Côté, 2008; White et al., 2011) which could be the result of small sample sizes evaluated for both. Further, our statistical analysis was limited by not evaluating potential random effects associated with years and individuals.

Although most western wildlife management agencies conduct predator control to protect ungulate populations this remains controversial (Reiter, Brunson & Schmidt, 1999). Predator removal is controversial and may only have short-term benefits for ungulate recovery (Reiter, Brunson & Schmidt, 1999; Hurley et al., 2011). Long-term solutions may include reductions of primary prey, which may benefit a rare or declining species by reducing limitation from puma. This was observed on the Patagonian steppe where the native guanaco (*Lama guanicoe*) increased when alternate prey (i.e., domestic sheep) were reduced in some areas (Novaro & Walker, 2005). In British Columbia, the reduction of the primary prey source of moose (*Alces alces*) through increased harvest in turn reduced the wolf population stopping the decline of an endangered woodland caribou population (Serrouya et al., 2017). Reducing the white-tailed deer population in areas near the core range of mountain goats may benefit this population in the long-term if puma remain the dominant predator in the Black Hills system. Additionally, vegetation management maintaining reduced tree density may decrease puma hiding cover (Lehman et al., 2017b) and reducing the amount of vegetation adjacent to precipitous escape terrain may allow mountain goats to better detect predators along movement corridors.

## Conclusions

Mountain goats can be highly susceptible to specialized predation when in small populations such as occurs in the Black Hills. A similar phenomena was observed for bighorn sheep in Alberta where the duration of predation events were stochastic and consistent with predation by specialist individuals (Festa-Bianchet et al., 2006); bighorn populations experienced one or two distinct puma predation events leading to population declines. Our research highlights the need for long-term studies of survival with a small, slow growing population as annual survival varied greatly over a 12-year period. If our research would have been conducted during shorter time periods (i.e., 2006–2010 or from 2011–2018) we would be reporting a much different result and associated inferences. It is difficult to disentangle the factors of puma predation and changing vegetation conditions in this system. Alternative hypotheses should be considered such as the potential for increased understory forage production potentially increasing mountain goat survival. Future research could continue to evaluate this relationship as ponderosa pine regeneration will presumably continue to increase woody vegetation density following the mountain pine beetle epidemic in the primary mountain goat range of the Black Hills. Additionally, monitoring the dynamics of puma predation as a specialist predator in this multi-prey system may provide insights into the stochastic nature of puma predation on mountain goats. Perhaps the death of one or two specialist pumas may explain the increases in mountain goat survival and future studies could benefit from examining these research hypotheses.

##  Supplemental Information

10.7717/peerj.9143/supp-1 Supplemental Information 1 Raw Data Mountain Goat Survival

## Additional Information and Declarations

### Data Availability

The following information was supplied regarding data availability: Raw data is available as a Supplemental File. Raw data also includes Program Mark results. Please contact the US Forest Service at: https://www.fs.usda.gov/about-agency/contact-us. Updated Geospatial Data can be accessed at: https://www.fs.usda.gov/main/blackhills/landmanagement/gis.
